# Cross sectional study of mode of delivery and maternal and perinatal outcomes in mainland China

**DOI:** 10.1371/journal.pone.0171779

**Published:** 2017-02-09

**Authors:** Lei Hou, Susan Hellerstein, Allison Vitonis, Liying Zou, Yan Ruan, Xin Wang, Weiyuan Zhang

**Affiliations:** 1 Department of Obstetrics, Beijing Obstetrics and Gynecology Hospital, Capital Medical University, Beijing, China; 2 Department of Obstetrics and Gynecology, Brigham and Women’s Hospital, Harvard Medical School, Boston MA, United States of America; Centre Hospitalier Universitaire Vaudois, FRANCE

## Abstract

**Background:**

Cesarean delivery (CD) rates have risen globally with nearly 50% of the non-indicated CDs worldwide in China and Brazil. In China’s One Child Policy era (1979–2015) most deliveries were women having their only child. Family size is a major determinant of the safety of medically non-indicated CD or CD on maternal request. The goal of this study is to document CD rates, indications, and analyze the relative safety of non-indicated CD compared to SVD and intrapartum CD.

**Methods:**

Univariate and multivariate logistic regression analyses of the association between mode of delivery and short-term maternal and perinatal outcomes were performed on a cross-section of all deliveries at 39 hospitals in 14 provinces of China in 2011, presented as adjusted odds ratio (aOR), 99% confidence intervals (CI).

**Findings:**

Among 108,847 deliveries, 59,415 were CD (54.6%) with 20.8% of deliveries or 38.2% of all cesareans were non-indicated CD. Compared to SVD, antepartum non-indicated CD was associated with a decreased likelihood of post-partum hemorrhage (PPH) (aOR = 0.80, CI = 0.69–0.92) and was not associated with maternal death or combined severe outcomes (maternal death, transfusion, or hysterectomy). Intrapartum indicated CD was associated with an increased risk of PPH (aOR = 1.68, CI = 1.50–1.89) compared to SVD. Compared to SVD, antepartum non-indicated CD was associated with lower likelihood of neonatal death (aOR = 0.14, CI = 0.06–0.34), neonatal ICU admission (aOR = 0.50, CI = 0.36–0.69), 5-minute Apgar<4 (aOR = 0.06, CI = 0.10–0.36), and respiratory distress syndrome (RDS) (aOR = 0.31, CI = 0.16–0.58), but not significantly associated with changes in rates of infection, hypoxic ischemic encephalopathy (HIEE), birth trauma or meconium aspiration rates.

**Conclusions:**

In 2011 when 81% of deliveries were women having their first child antepartum non-indicated CD had short-term maternal and perinatal outcomes as safe as SVD. Now that all Chinese women can have a second child the safety profile may change.

## Introduction

Cesarean delivery (CD) rates have risen rapidly globally [1,2] with nearly 50% of the world’s non-indicated CD [3] done in China and Brazil. Expectant women and families consider safety of delivery critical in decision-making. Chinese obstetricians experience tremendous pressure to produce a “perfect” baby. The relative safety of modes of delivery needs elucidation [4].

A 2008 World Health Organization (WHO) analysis reported a CD rate of 46% in China[1]. Although not officially available, Chinese studies report national CD rates from 36% to 58% [5,6]. Non-indicated CD, including cesarean delivery on maternal request (CDMR) and other indications not recognized internationally, accounts for a large portion of China’s high CD rate. Guidelines on CDMR from the United States of America (USA) [7] and United Kingdom (UK) [8] both define CDMR as a primary pre-labor cesarean delivery on maternal request without fetal or maternal indication. In China, the definition of CDMR is less clear and studies use the terms CD on maternal request and CD for “social influence” variably for non-indicated CD. A “CDMR” diagnosis may reflect an explicit or implicit provider recommendation [9].

The CDMR rate is estimated at 2.5–3% of births in the USA[10] and 1–2% [11] in the UK. In a study of 56,968 CDs in China the prevalence of primary non-indicated CD increased from 0.6% of deliveries in 1993–5 to 12.9% in 2001–5 [12]. This is consistent with a 10% non-indicated CD rate in a study of 1.3 million deliveries [13] and 11.6% in the 2008 WHO [1]. However, a meta-analysis of 49 papers published in Mandarin from 2000–2009 found that 27.1% of CDs were done for “social influence”[5].

The National Institutes of Health (NIH) conference on CDMR literature review, which informed the USA[7] and UK[8] guidelines, found in term vertex singleton pregnancies, CDMR is associated with a lower rate of maternal hemorrhage, a longer maternal inpatient stay, higher rates of neonatal respiratory morbidity (especially in CD done prior to 39 weeks), and greater complications in subsequent pregnancies, such as uterine rupture, placenta previa, placenta accreta, bladder and bowel injuries, and the need for hysterectomy[10]. Since intended family size influences the safety profile of cesareans, CDMR is not recommended for women desiring “several children”[7,8].

China’s One Child Policy (1979–2015) created an era of nulliparous maternity care with most women delivering their only child, which could influence the relative safety of non-indicated CD. With the large number of non-indicated CD in China, analysis of the association between mode of delivery and maternal and neonatal outcomes in China can be compared with international studies on CDMR.

This study aims to document CD rates, indications for CD, and to study the associations between mode of delivery and short-term maternal and perinatal outcomes in China with a focus on non-indicated CD.

## Methods and materials

Discharge data was collected from all births greater than 24 weeks gestation from January 1, 2011 through December 31, 2011 in 39 public hospitals from 14 provinces of Mainland China (Beijing, Shanghai, Jilin, Liaoning, Jiangsu, Sichuan, Shanxi, Hubei, Guangdong, Hebei, Inner Mongolia, Shandong, Shanxi, and Xinjiang). These participating hospitals are members of an obstetrics cooperative center with broader medical and academic collaboration. All hospitals were secondary or tertiary care public hospitals because in 2011 more than 95% of deliveries occurred at public hospitals and less than 1% of deliveries occurred at primary care facilities [14,15]. A physician trained in obstetrics was present at all deliveries.

The individual-level data obtained from medical records was coded in a de-identified format under waiver of consent. Institutional informed consent was obtained from the responsible authority at each of the participating health facilities in China (S1 Appendix). The physician coordinator at each site was trained on data extraction. Data was extracted from medical records and discharge summaries by trained medical staff on a standardized coded form, and then entered for computer-based statistical analysis.

Data points included: demographics, maternal data (age, parity, education, medical comorbidities, obesity), obstetric factors (gestational age, presentation, gestational diabetes, preeclampsia, premature rupture of membranes, third trimester bleeding), mode of delivery, indication for CD, and antepartum or intrapartum timing of the CD. Obesity in China is defined as body mass index (BMI)≥28 [16]. The physician-documented indication for CD was recorded. If there was more than one indication the physician designated primary indication was used. Short-term maternal outcomes from the birth admission included: post partum hemorrhage (PPH) (defined in China as the loss of more than 500 mL of blood during the first 24 hours after any delivery), inpatient maternal death, blood transfusion, hysterectomy during the birth admission, wound dehiscence, tear of uterine angle, puerperal infection, and venous thromboembolism (VTE). Perinatal outcomes included: neonatal death (live birth with death in the first 7 days), perinatal death (defined as stillbirths after 24 weeks gestation plus neonatal deaths), neonatal ICU admission, Apgar scores, respiratory distress syndrome (RDS), infection, hypoxic ischemic encephalopathy (HIEE), birth trauma, and meconium aspiration.

The dataset did not include information about the severity of PPH (using estimated blood loss greater than 1000 or 1500 ml), length of stay, re-admission data, income level, or insurance status.

The dataset included 109,806 deliveries great than 24 weeks gestation. All births designated as terminations were excluded (n = 458). Additionally all stillbirths (n = 501) were excluded because it was not possible to distinguish between antenatal and intrapartum fetal demise. The final sample included 108,847 deliveries. Vaginal birth, operative vaginal birth, and overall CD rates were calculated overall and for each region. Operative vaginal birth was defined as forceps or vacuum assisted vaginal delivery.

CDs were divided into two categories, indicated and non-indicated. The indicated CD category included: previous CD, non-reassuring fetal heart tracing (NRFHT), arrest of labor (failure to progress or cephalo-pelvic-disproportion (CPD) in labor, malpresentation, and previous uterine surgery. NRFHT criteria were consistent with definitions in Williams Obstetrics (23rd Edition). In 2011, vaginal birth after cesarean (VBAC) was not offered in China. There are other common indications in China which are not necessarily globally accepted indications for CD, including; preeclampsia/eclampsia/HELLP, oligohydramnios, third trimester bleeding (previa/accrete/abruption placenta), multiple gestation, suspected macrosomia, and others (for indications with low frequencies). These were included in the “indicated” category in this study because there was not sufficient clinical information in the database to determine which of these cases would meet internationally accepted criteria. For example, the severity of preeclampsia, the estimated fetal weight, details of third trimester bleeding or of multiple pregnancies (higher order pregnancies, twin presentation and growth concordance) were unknown. Criteria for some of these indications are based on the accepted obstetric standards in China. For example, suspected macrosomia was an estimated fetal weight (EFW) >4000 grams, based on ultrasound or Leopold’s maneuvers, regardless of diabetic status. Fetal growth restriction was a fetus with an estimated birth weight less than the 10th percentile.

A non-indicated CD category was defined as a primary CD documented by the physician done on “maternal request” in the absence of maternal or fetal indication or physician documented reason that shows a provider preference but not an internationally recognized indication. These included: cephalo-pelvic disproportion prior to the onset of labor based on clinical pelvimetry and/or EFW, maternal age 35 or older as the only indication, “precious” fetus-defined as in vitro pregnancy or poor obstetric history (i.e. prior fetal death, neonatal death, chromosomal or structural abnormality), isolated premature rupture of membranes without fetal heart rate (FHR) abnormalities, nuchal cord seen on ultrasound without FHR abnormalities, severe myopia, request for concomitant myomectomy or ovarian cystectomy, or other (isolated chronic hypertension; gestational hypertension; diabetes mellitus without macrosomia).

We examined mode of delivery by region and by maternal characteristics using descriptive statistics. Logistic regression models were used to estimate the association between mode of delivery and dichotomous maternal and neonatal outcomes. Results of the logistic regression analyses were expressed as crude and adjusted odds ratios (aORs) with 99% confidence intervals (CIs). All models were adjusted *a priori* for maternal age (continuous), education (college or higher, high school, primary school, illiterate), gestational weeks (continuous), malpresentation (yes, no), parity (continuous), multiple gestation (yes, no), placenta previa (yes, no), placenta abruption (yes, no), prebirth bleeding (yes, no), malformations (yes, no), and medical complications (yes, no). Missing values for adjustment variables were handled by the missing indicator method [17]. Outcome variables with missing data included maternal death (n = 16), surgical complication (defined as wound dehiscence or tear of UT angle) (n = 8), neonatal death (n = 38), perinatal death (n = 31), admission to neonatal ICU (n = 38), 5 minute Apgar score (n = 4,399), and neonatal complication (IRDS, infection, HIEE/cerebral hemorrhage, birth trauma, meconium aspiration, n = 36). We conducted a sensitivity analysis in which we repeated our logistic regression analysis after restricting the sample to primiparous women with singleton deliveries and excluded multiples and those with malpresentation and malformations (n = 79,110). Analyses were performed using SAS 9.3. All P values were two-sided, and statistical significance was defined as p<0.01.

## Results

**Fig 1:**
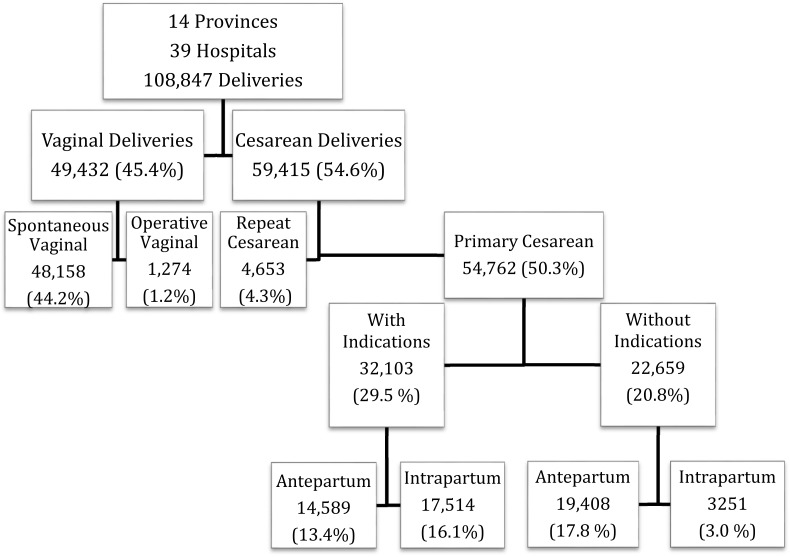
Study design and cesarean delivery rates.

A total of 108,847deliveries over 24 weeks gestation were analyzed in this study. There were 59,415 CD for an overall CD rate of 54.6%. 54,762 CD (92.2% of CD or 50.3% of deliveries) were primary CD and 4,653 (7.8% of CD or 4.3% of deliveries) were repeat CD (Fig 1 and Table 1).

**Table 1:** Vaginal and cesarean (CD) delivery rates for 39 hospitals in 3 geographical regions across China.

Region	Indicated CD		Non-indicated CD		Overall CD	Vaginal delivery		Overall Vaginal	Total number of deliveries
	Antepartum	Intrapartum	Antepartum	Intrapartum		Spontaneous	Operative*		
	N (%)	N (%)	N (%)	N (%)	N (%)	N (%)	N (%)	N (%)	N
Eastern	13126 (19.2)	9514 (13.9)	11313 (16.5)	1238 (1.8)	35191 (51.5)	32167 (47.1)	1008 (1.5)	33175 (48.5)	68366
Central	3319 (20.4)	1797 (11.1)	4992 (30.7)	543 (3.3)	10651 (65.6)	5443 (33.5)	142 (0.9)	5585 (34.4)	16236
Western	2797 (11.5)	6203 (25.6)	3103 (12.8)	1470 (6.1)	13573 (56.0)	10548 (43.5)	124 (0.5)	10672 (44.0)	24245
Total	19242	17514	19408	3251	59415	48158	1274	49432	108847

* Operative vaginal delivery includes forceps delivery, vacuum extraction delivery, breech delivery and breech extraction.

Overall, 38,650 (65.0%) of CDs were performed antepartum, prior to the onset of labor. 22,659 CD (38.1% of CD or 20.8% of deliveries) were non-indicated. Of the non-indicated CDs, 19,408 (85.7%) were planned and performed prior to the onset of labor. There were only 2 vaginal births after cesarean (VBAC) in the dataset. (Fig 1).

### Population characteristics

The characteristics of women by mode of delivery are presented in Table 2. Overall, 81.4% of all deliveries were women having their first child. The mean maternal age at delivery was 28.2. Women 35 years old or greater had a 70.0% CD rate. Male infants accounted for 54.6% of all deliveries. Overall 17.4% of women were obese with a BMI≥ 28 [16].

**Table 2:** Characteristics of the study population by mode of delivery.

	Indicated CD		Non-indicated CD		Overall CS	Vaginal delivery		Overall Vaginal	Total number of
	Antepartum	Intrapartum	Antepartum	Intrapartum		Spontaneous	Operative		deliveries
	N (%)	N (%)	N (%)	N (%)	N (%)	N (%)	N (%)	N (%)	N (%)
Primiparous									
No	5202 (27.0)	3381 (19.3)	1819 (9.4)	222 (6.8)	10624 (17.9)	9571 (19.9)	92 (7.2)	9663 (19.5)	20287 (18.6)
Yes	14040 (73.0)	14133 (80.7)	17589 (90.6)	3029 (93.2)	48791 (82.1)	38587 (80.1)	1182 (92.8)	39769 (80.5)	88560 (81.4)
Maternal age									
<21	401 (2.1)	435 (2.5)	645 (3.4)	50 (1.5)	1531 (2.6)	1815 (3.8)	23 (1.8)	1838 (3.7)	3369 (3.1)
21–34	15955 (83.6)	15100 (86.7)	16149 (84.0)	2637 (81.6)	49841 (84.5)	42851 (89.6)	1160 (91.4)	44011 (89.6)	93852 (86.9)
>34	2729 (14.3)	1885 (10.8)	2425 (12.6)	543 (16.8)	7582 (12.9)	3172 (6.6)	86 (6.8)	3258 (6.6)	10840 (10.0)
Mean (SD)	29.2 (4.9)	28.6 (4.7)	28.4 (5.0)	29.5 (4.8)	28.8 (4.9)	27.5 (4.4)	28.7 (3.9)	27.5 (4.4)	28.2 (4.7)
Education									
College or higher	9962 (53.6)	8335 (49.3)	9911 (52.2)	1906 (60.4)	30114 (52.2)	23680 (51.5)	752 (60.1)	24432 (51.7)	54546 (52.0)
High school	5225 (28.1)	4555 (27.0)	5522 (29.1)	777 (24.6)	16079 (27.9)	12495 (27.2)	366 (29.2)	12861 (27.2)	28940 (27.6)
Primary school	3363 (18.1)	3950 (23.4)	3520 (18.5)	468 (14.8)	11301 (19.6)	9697 (21.1)	134 (10.7)	9831 (20.8)	21132 (20.2)
Illiterate	44 (0.2)	51 (0.3)	45 (0.2)	5 (0.2)	145 (0.3)	101 (0.2)	0 (0)	101 (0.2)	246 (0.2)
Hospital Level									
Secondary	4849 (25.2)	3546 (20.2)	7346 (37.9)	732 (22.5)	16473 (27.7)	15639 (32.5)	117 (9.2)	15756 (31.9)	32229 (29.6)
Tertiary	14393 (74.8)	13968 (79.8)	12062 (62.1)	2519 (77.5)	42942 (72.3)	32519 (67.5)	1157 (90.8)	33676 (68.1)	76618 (70.4)
Resident local									
Migrant	6252 (32.5)	5538 (31.6)	6139 (31.6)	611 (18.8)	18540 (31.2)	14426 (30.0)	471 (37.0)	14897 (30.1)	33437 (30.7)
Local	12990 (67.5)	11976 (68.4)	13269 (68.4)	2640 (81.2)	40875 (68.8)	33732 (70.0)	803 (63.0)	34535 (69.9)	75410 (69.3)
Obesity									
No	11472 (73.1)	11791 (79.2)	12936 (81.8)	1899 (79.6)	38098 (78.1)	35768 (87.8)	978 (87.3)	36746 (87.8)	74844 (82.6)
Yes	4228 (26.9)	3089 (20.8)	2884 (18.2)	487 (20.4)	10688 (21.9)	4954 (12.2)	142 (12.7)	5096 (12.2)	15784 (17.4)
Bad OB history									
No	19038 (98.9)	17273 (98.6)	19297 (99.4)	3219 (99.0)	58827 (99.0)	47784 (99.2)	1265 (99.3)	49049 (99.2)	107876 (99.1)
Yes	204 (1.1)	241 (1.4)	111 (0.6)	32 (1.0)	588 (1.0)	374 (0.8)	9 (0.7)	383 (0.8)	971 (0.9)
Male fetus									
No	8542 (44.4)	7888 (45.0)	8569 (44.2)	1407 (43.3)	26406 (44.4)	22511 (46.7)	527 (41.4)	23038 (46.6)	49444 (45.4)
Yes	10700 (55.6)	9626 (55.0)	10839 (55.8)	1844 (56.7)	33009 (55.6)	25647 (53.3)	747 (58.6)	26394 (53.4)	59403 (54.6)
Multiple gestation									
No	18230 (94.7)	16890 (96.4)	19408 (100.0)	3251 (100.0)	57779 (97.2)	47932 (99.5)	1266 (99.4)	49198 (99.5)	106977 (98.3)
Yes	1012 (5.3)	624 (3.6)	0 (0)	0 (0)	1636 (2.8)	226 (0.5)	8 (0.6)	234 (0.5)	1870 (1.7)
GDM, DM									
No	17970 (93.4)	16550 (94.5)	18314 (94.4)	2976 (91.5)	55810 (93.9)	46524 (96.6)	1209 (94.9)	47733 (96.6)	103543 (95.1)
Yes	1272 (6.6)	964 (5.5)	1094 (5.6)	275 (8.5)	3605 (6.1)	1634 (3.4)	65 (5.1)	1699 (3.4)	5304 (4.9)
HDCP									
No	16937 (88.0)	15850 (90.5)	19146 (98.7)	3209 (98.7)	55142 (92.8)	47324 (98.3)	1227 (96.3)	48551 (98.2)	103693 (95.3)
Yes	2305 (12.0)	1664 (9.5)	262 (1.3)	42 (1.3)	4273 (7.2)	834 (1.7)	47 (3.7)	881 (1.8)	5154 (4.7)
Eclampsia									
No	17217 (89.5)	16112 (92.0)	19408 (100.0)	3251 (100.0)	55988 (94.2)	47658 (99.0)	1242 (97.5)	48900 (98.9)	104888 (96.4)
Yes	2025 (10.5)	1402 (8.0)	0 (0)	0 (0)	3427 (5.8)	500 (1.0)	32 (2.5)	532 (1.1)	3959 (3.6)
PROM									
No	17912 (93.1)	13998 (79.9)	17616 (90.8)	2395 (73.7)	51921 (87.4)	39135 (81.3)	939 (73.7)	40074 (81.1)	91995 (84.5)
Yes	1330 (6.9)	3516 (20.1)	1792 (9.2)	856 (26.3)	7494 (12.6)	9023 (18.7)	335 (26.3)	9358 (18.9)	16852 (15.5)
Preterm delivery									
No	17336 (90.1)	15289 (87.3)	18996 (97.9)	3067 (94.3)	54688 (92.0)	44675 (92.8)	1181 (92.7)	45856 (92.8)	100544 (92.4)
Yes	1906 (9.9)	2225 (12.7)	412 (2.1)	184 (5.7)	4727 (8.0)	3483 (7.2)	93 (7.3)	3576 (7.2)	8303 (7.6)
Prebirth bleeding									
No	18200 (94.6)	16489 (94.1)	19408 (100.0)	3251 (100.0)	57348 (96.5)	47949 (99.6)	1257 (98.7)	49206 (99.5)	106554 (97.9)
Yes	1042 (5.4)	1025 (5.9)	0 (0)	0 (0)	2067 (3.5)	209 (0.4)	17 (1.3)	226 (0.5)	2293 (2.1)
Oligohydrammios									
No	17669 (91.8)	16740 (95.6)	19408 (100.0)	3251 (100.0)	57068 (96.0)	48158 (100.0)	1274 (100.0)	49432 (100.0)	106500 (97.8)
Yes	1573 (8.2)	774 (4.4)	0 (0)	0 (0)	2347 (4.0)	0 (0)	0 (0)	0 (0)	2347 (2.2)
Gestational age									
Mean (SD)	38.7 (2.2)	38.8 (2.4)	39.3 (1.6)	38.9 (1.7)	38.9 (2.1)	39.2 (2.2)	39.2 (2.6)	39.2 (2.3)	39.0 (2.2)
Birthweight									
≤4000 g	15957 (83.7)	15710 (91.4)	18665 (97.1)	3168 (98.5)	53500 (91.1)	45411 (95.9)	1182 (94.1)	46593 (95.8)	100093 (93.3)
4000 g	3113 (16.3)	1475 (8.6)	565 (2.9)	48 (1.5)	5201 (8.9)	1964 (4.1)	74 (5.9)	2038 (4.2)	7239 (6.7)
Malformation									
No	19213 (99.9%)	17466 (99.8%)	19388 (99.9%)	3247 (99.9%)	59314 (99.9%)	48022 (99.8%)	1268 (99.5%)	49290 (99.8%)	108604 (99.8)
Yes	25 (0.1%)	42 (0.2%)	15 (0.1%)	3 (0.1%)	85 (0.1%)	114 (0.2%)	6 (0.5%)	120 (0.2%)	205 (0.2)

Women who had CD compared to SVD tended to be older (12.9% vs. 6.6%) and with medical or obstetric factors such as obesity (21.9% vs. 12.2%), hypertensive disease (7.2% vs. 1.7%), pre-birth bleeding (3.5% vs. 0.4%), oligohydramios (4.0% vs. 0%) and birth weight >4000 grams (8.9% vs. 4.1%). The mean gestational age for SVD, all CD and non-indicated CD was 39.2, 38.9 and 39.3 weeks, respectively. 27% of SVD and non-indicated CD deliveries were less than 39 weeks gestation. Of the non-indicated antepartum CD, 72.9% were done at or after 39 weeks, 25% from 37–38 weeks, 1.38% 34–36 weeks and 0.52% less than 34 weeks (data not shown).

A greater percentage of women delivering by non-indicated antepartum CD were having their first child (90.6%) compared to those with a SVD (80.1%) or those with an indicated antepartum CD (73.0%) Additionally women with non-indicated antepartum CD compared to SVD were more likely to be over 34 years old (12.6% vs. 6.6%), obese (18.2% vs. 12.2%), and with diabetes (5.6% vs. 3.4%).

Women delivering with indicated antepartum CD compared to SVD were more likely to be obese (26.9% vs. 12.2%), have antepartum hemorrhage (5.4% vs. 0.4%), hypertensive disease (12.0% vs. 1.7%), eclampsia (10.5% vs. 1.0%), GDM (6.6% vs. 3.4%), or multiple pregnancy (5.3% vs. 0.5%). Women with PROM were more likely to have a vaginal delivery.

Women with indicated intrapartum CD compared to SVD were similar in parity (80.7% vs. 80.1%) and college education (49.3% vs. 51.5%), but more likely to be over 34 years old (10.8% vs. 6.6%), obese (20.8% vs. 12.2%), have an infant weighing >4000 grams (8.6% vs. 4.1%) or with other high risk obstetric issues such as hypertensive disease (9.5% vs.1.7%), pre birth bleeding (5.9% vs.0.4%), multiple gestation (3.6% vs. 0.5%), or oligohydramnios (4.4% vs. 0%).

### Indications for cesarean delivery

Among the 59,415 CDs, the 6 most commonly recorded indications were: “maternal request” (n = 13,778, 23.2%), NRFHRT (n = 7603, 12.8%), FTP/CPD in labor (n = 5275, 8.9%), repeat CD (n = 4653, 7.8%), “CPD” diagnosed antepartum (n = 4646, 7.8%) and malpresentation (n = 3448, 5.8%) (Table 3).

**Table 3:** Indications for cesarean delivery in China.

	N	% of all Cesarean Deliveries	% of all Deliveries
**Total number of cesarean deliveries**	59415		54.6%
** Repeat**	4653	7.8%	4.3%
** Primary**	54762	92.2%	50.3%
** Non-indicated primary CD**	22659		
Maternal request	13778	23.2%	12.7%
CPD diagnosed antepartum	4646	7.8%	4.3%
Other non-indicated CD	4235	7.1%	3.9%
** Indicated primary CD**	32103		
Non-reassuring fetal testing	7603	12.8%	7.0%
Failure to progress	5275	8.9%	4.8%
Suspected macrosomia	3370	5.7%	3.1%
Malpresentation	3448	5.8%	3.2%
Preeclampsia/eclampsia	2632	4.4%	2.4%
Oligohydrammios	2347	4.0%	2.2%
Late pregnancy bleeding^*^	1597	2.7%	1.5%
Previous uterine surgery	1496	2.5%	1.4%
Multiple gestation	1265	2.1%	1.2%
Other	3070	5.2%	2.8%

* Late pregnancy bleeding includes-placenta previa, accreta and abruption placenta.

Among the 22,659 non-indicated CD, CDMR was the most common diagnosis (n = 13,778, 60.8%), followed by CPD prior to labor (n = 4646, 20.5%), and age ≥35 years (n = 2591, 11.4%). Other coded reasons included: “precious baby” (defined as an in vitro fertilization pregnancy or a patient with a prior loss), request for myomectomy, nuchal cord, isolated PROM, and severe myopia.

The overall CD rates varied in the three geographical regions, with 65.6% in Central China, 56.0% in Western China, and 51.5% in Eastern China (Table 1). The proportion of women with non-indicated CD varied across geographic region, ranging from 18% to 34% of all deliveries, with the highest rate also observed in Central China. Regional differences in total CD rates were driven by variation in CD for CDMR and CPD prior to labor, rather than differences in parity, maternal age, education, or indicated CD (data not shown).

### Outcomes

#### Maternal (Table 4)

**Table 4:** Associations between mode of delivery and maternal outcomes.

Mode of delivery	No outcome	Outcome	Crude	Adjusted*	Adjusted*
	N (%)	N (%)	OR (99% CI)	OR (99% CI)	p-value
Outcome: Inpatient maternal death					
Spontaneous	48147 (44.2%)	3 (42.9%)	1.00		
Operative vaginal delivery	1274 (1.2%)	0 (0%)	—		
Non-Indicated antepartum	19406 (17.8%)	0 (0%)	—		
Non-Indicated intrapartum	3250 (3.0%)	0 (0%)	—		
Indicated antepartum	19238 (17.7%)	3 (42.9%)	2.50 (0.31, 20.5)		
Indicated intrapartum	17509 (16.1%)	1 (14.3%)	0.92 (0.05, 18.0)		
Outcome: Postpartum hemorrhage					
Spontaneous	46813 (44.7%)	1345 (32.6%)	1.00	1.00	
Operative vaginal delivery	1180 (1.1%)	94 (2.3%)	2.77 (2.09, 3.69)	2.49 (1.86, 3.32)	<0.0001
Non-Indicated antepartum	18961 (18.1%)	447 (10.8%)	0.82 (0.71, 0.95)	0.80 (0.69, 0.92)	<0.0001
Non-Indicated intrapartum	3125 (3.0%)	126 (3.1%)	1.40 (1.10, 1.79)	1.30 (1.01, 1.66)	0.007
Indicated antepartum	18228 (17.4%)	1014 (24.6%)	1.94 (1.74, 2.16)	1.28 (1.13, 1.45)	<0.0001
Indicated intrapartum	16412 (15.7%)	1102 (26.7%)	2.34 (2.10, 2.60)	1.68 (1.50, 1.89)	<0.0001
Outcome: Maternal death, hysterectomy, transfusion, or VTE					
Spontaneous	48022 (44.4%)	136 (21.1%)	1.00	1.00	
Operative vaginal delivery	1262 (1.2%)	12 (1.9%)	3.36 (1.54, 7.32)	3.15 (1.42, 6.99)	0.0002
Non-Indicated antepartum	19363 (17.9%)	45 (7.0%)	0.82 (0.53, 1.28)	0.94 (0.60, 1.47)	0.73
Non-Indicated intrapartum	3234 (3.0%)	17 (2.6%)	1.86 (0.96, 3.61)	2.05 (1.05, 4.00)	0.006
Indicated antepartum	19076 (17.6%)	166 (25.7%)	3.07 (2.28, 4.14)	1.35 (0.96, 1.88)	0.02
Indicated intrapartum	17244 (15.9%)	270 (41.8%)	5.53 (4.21, 7.26)	2.54 (1.88, 3.43)	<0.0001

*Adjusted for maternal age and education, gestational weeks, malpresentation, parity, multiple, placenta previa, placenta abruption, prebirth bleeding, medical complications, and malformation.

Among all 108,847 deliveries, physician diagnosed postpartum hemorrhage occurred in 4128 (4%) of births. Surgical complications, including extension of the uterine incision (46) or wound dehiscence (n = 415), occurred in 461 (0.8%) of CD. There were 627 (0.6%) women with blood transfusions, 39 hysterectomies (0.04%) and 7 cases with recorded venous thromboembolism (VTE). There were 7-inpatient maternal deaths for a maternal mortality rate of 6.4/100,000 (7/108,847).

Compared with SVD, non-indicated antepartum CD was associated with lower risk of PPH (aOR = 0.80, CI = 0.69–0.92) while all other modes of delivery were associated with increased risk PPH risk. Compared to SVD there was no association between non-indicated antepartum CD and combined severe outcomes (death, transfusion, hysterectomy, VTE) (aOR = 0.94, CI = 0.60–1.47). Compared to SVD operative vaginal and intrapartum cesareans delivery types were associated with increased risk of a severe maternal outcome.

Analysis of all cesareans showed women with either indicated or non-indicated intrapartum CD compared to non-indicated antepartum CD were significantly more likely to experience a severe maternal outcome (death, transfusion, hysterectomy, or VTE) (aOR for indicated intrapartum = 2.56, CI = 1.88–3.43); aOR for non-indicated intrapartum = 2.05, CI = 1.05–4.05) (data not shown), even after multivariable adjustment. There were no associations between CD type and surgical complications comparing non-indicated antepartum CD to all other types of CD.

#### Perinatal outcomes (Table 5)

**Table 5:** Associations between mode of delivery and neonatal outcomes.

Mode of delivery	No outcomeN (%)	OutcomeN (%)	CrudeOR (99% CI)	Adjusted*OR (99% CI)	Adjusted* p-value
Outcome: Neonatal death					
Spontaneous	47814 (44.1%)	322 (76.8%)	1.00	1.00	
Operative vaginal delivery	1260 (1.2%)	14 (3.3%)	1.65 (0.81, 3.35)	0.94 (0.37, 2.42)	0.87
Non-Indicated antepartum	19394 (17.9%)	9 (2.1%)	0.07 (0.03, 0.17)	0.14 (0.06, 0.34)	<0.0001
Non-Indicated intrapartum	3250 (3.0%)	0 (0%)	—	—	
Indicated antepartum	19189 (17.7%)	49 (11.7%)	0.38 (0.26, 0.56)	0.19 (0.12, 0.31)	<0.0001
Indicated intrapartum	17483 (16.1%)	25 (6.0%)	0.21 (0.12, 0.36)	0.10 (0.05, 0.18)	<0.0001
Outcome: Admission to neonatal ICU					
Spontaneous	47569 (44.2%)	567 (50.5%)	1.00	1.00	
Operative vaginal delivery	1244 (1.2%)	30 (2.7%)	2.02 (1.24, 3.30)	2.87 (1.71, 4.80)	<0.0001
Non-Indicated antepartum	19326 (17.9%)	77 (6.9%)	0.33 (0.24, 0.46)	0.50 (0.36, 0.69)	<0.0001
Non-Indicated intrapartum	3225 (3.0%)	25 (2.2%)	0.65 (0.38, 1.10)	0.83 (0.48, 1.42)	0.36
Indicated antepartum	19066 (17.7%)	172 (15.3%)	0.76 (0.60, 0.95)	0.73 (0.57, 0.94)	0.001
Indicated intrapartum	17256 (16.0%)	252 (22.4%)	1.23 (1.01, 1.49)	1.18 (0.95, 1.47)	0.05
Outcome: Low Apgar(5min Apgar<4)					
Spontaneous	45658 (43.8%)	172 (69.9%)	1.00	1.00	
Operative vaginal delivery	1203 (1.2%)	11 (4.5%)	2.43 (1.09, 5.43)	1.64 (0.59, 4.58)	0.21
Non-Indicated antepartum	18655 (17.9%)	2 (0.8%)	0.03 (0.01, 0.18)	0.06 (0.01, 0.36)	<0.0001
Non-Indicated intrapartum	3209 (3.1%)	0 (0%)	—	—	
Indicated antepartum	18470 (17.7%)	29 (11.8%)	0.42 (0.25, 0.70)	0.25 (0.14, 0.46)	<0.0001
Indicated intrapartum	17007 (16.3%)	32 (13.0%)	0.50 (0.30, 0.82)	0.28 (0.16, 0.50)	<0.0001
Outcome: Respiratory Distress (IRDS)					
Spontaneous	47925 (44.2%)	212 (45.7%)	1.00	1.00	
Operative vaginal delivery	1252 (1.2%)	22 (4.7%)	3.97 (2.22, 7.11)	4.09 (2.18, 7.67)	<0.0001
Non-Indicated antepartum	19386 (17.9%)	18 (3.9%)	0.21 (0.11, 0.40)	0.31 (0.16, 0.58)	<0.0001
Non-Indicated intrapartum	3246 (3.0%)	4 (0.9%)	0.28 (0.08, 1.02)	0.35 (0.09, 1.27)	0.04
Indicated antepartum	19143 (17.7%)	95 (20.5%)	1.12 (0.82, 1.54)	0.94 (0.66, 1.34)	0.66
Indicated intrapartum	17395 (16.1%)	113 (24.4%)	1.47 (1.09, 1.98)	1.22 (0.88, 1.69)	0.12
Outcome: Infection					
Spontaneous	48108 (44.2%)	29 (34.9%)	1.00	1.00	
Operative vaginal delivery	1273 (1.2%)	1 (1.2%)	1.30 (0.10, 17.9)	0.83 (0.05, 12.9)	0.86
Non-Indicated antepartum	19398 (17.8%)	6 (7.2%)	0.51 (0.16, 1.63)	0.70 (0.22, 2.26)	0.43
Non-Indicated intrapartum	3248 (3.0%)	2 (2.4%)	1.02 (0.16, 6.72)	1.21 (0.18, 8.06)	0.80
With-indication antepartum	19219 (17.7%)	19 (22.9%)	1.64 (0.77, 3.51)	1.41 (0.61, 3.26)	0.29
With-indication intrapartum	17482 (16.1%)	26 (31.3%)	2.47 (1.23, 4.95)	1.99 (0.94, 4.21)	0.02
Outcome: HIEE/cerebral hemorrhage					
Spontaneous	48121 (44.2%)	16 (57.1%)	1.00	1.00	
Operative vaginal delivery	1272 (1.2%)	2 (7.1%)	4.73 (1.09, 20.6)	7.18 (1.75, 68.8)	0.02
Non-Indicated antepartum	19404 (17.8%)	0 (0%)	—	—	
Non-Indicated intrapartum	3249 (3.0%)	1 (3.6%)	0.93 (0.12, 6.98)	1.67 (0.12, 24.2)	0.62
Indicated antepartum	19234 (17.7%)	4 (14.3%)	0.63 (0.21, 1.87)	0.67 (0.14, 3.28)	0.52
Indicated intrapartum	17503 (16.1%)	5 (17.9%)	0.86 (0.32, 2.35)	0.82 (0.20, 3.40)	0.71
Outcome: Birth Trauma					
Spontaneous	48116 (44.2%)	21 (65.6%)	1.00	1.00	
Operative vaginal delivery	1272 (1.2%)	2 (6.3%)	4.73 (0.68, 32.7)	6.97 (0.73, 66.5)	0.03
Non-Indicated antepartum	19402 (17.8%)	2 (6.3%)	—	—	—
Non-Indicated intrapartum	3250 (3.0%)	0 (0%)	—	—	—
Indicated antepartum	19234 (17.7%)	4 (12.5%)	0.63 (0.15, 2.64)	0.66 (0.14, 3.21)	0.50
Indicated intrapartum	17505 (16.1%)	3 (9.4%)	0.86 (0.23, 3.22)	0.81 (0.19, 3.36)	0.70
Outcome: Meconium aspiration					
Spontaneous	48046 (44.2%)	91 (40.8%)	1.00	1.00	
Operative vaginal delivery	1269 (1.2%)	5 (2.2%)	2.08 (0.64, 6.81)	2.60 (0.78, 8.65)	0.04
Non-Indicated antepartum	19385 (17.9%)	19 (8.5%)	0.52 (0.27, 0.99)	0.66 (0.34, 1.27)	0.10
Non-Indicated intrapartum	3244 (3.0%)	6 (2.7%)	0.98 (0.33, 2.90)	1.16 (0.39, 3.47)	0.73
Indicated antepartum	19209 (17.7%)	29 (13.0%)	0.80 (0.46, 1.38)	0.79 (0.44, 1.42)	0.29
Indicated intrapartum	17435 (16.1%)	73 (32.7%)	2.21 (1.47, 3.32)	2.15 (1.40, 3.31)	<0.0001

*Adjusted for maternal age and education, gestational weeks, malpresentation, parity, multiple, placenta previa, placenta abruption, prebirth bleeding, medical complications, and malformation.

Among all deliveries there were 8303 (7.6%) preterm deliveries (<37 weeks), 1123 (1%) of newborns admitted to the ICU, 246 (0.2%) with a 5 minute Apgar < 4, 464 (0.4%) cases of respiratory distress syndrome, 223 (0.2%) cases of meconium aspiration syndrome, 83 newborns with severe perinatal infection, 32 with birth trauma, and 28 with HIEE/cerebral hemorrhage. There were 419 neonatal deaths. The perinatal mortality rate was not calculated because of the inability to distinguish presentation with stillbirth from intrapartum stillbirth. The early (0–7 days) neonatal mortality rate was 3.85/1000 live births.

Compared with SVD, antepartum non-indicated CD was associated with decreased risk of neonatal death (aOR = 0.14, CI = 0.06–0.34), admission to ICU (aOR = 0.50, CI = 0.36–0.69), 5-min Apgar of less than four (aOR = 0.06, CI = 0.01–0.36), and respiratory distress syndrome (aOR = 0.31, CI = 0.16–0.58). Rates of infection, hypoxic ischemic encephalopathy, or meconium aspiration did not significantly differ between SVD and antepartum non-indicated CD.

Indicated intrapartum CD was associated with decreased likelihood of neonatal death (aOR = 0.10, CI = 0.05–0.18) and 5 min Apgar less than four (aOR = 0.28, CI = 0.16–0.50), no change in admission to NICU, RDS, and infection and an increased risk of meconium aspiration (aOR = 2.15, CI = 1.40–3.31), compared to delivery by SVD.

Operative vaginal deliveries were associated with higher risk of PPH (aOR = 2.49, CI = 1.86–3.32) combined severe maternal outcomes (aOR = 3.15, CI = 1.42–6.99), neonatal ICU admission (aOR = 2.87, CI = 1.71–4.80), and RDS (aOR = 4.09, CI = 2.18–7.67) compared to SVD.

We examined the same associations between mode of delivery and maternal and perinatal outcomes in a sensitivity analysis in which we excluded multiparous women, multiple gestations, mal-presentations, malformations, and preterm births. In the restricted analysis of nulliparous, singleton, vertex, term births, without malformations, the findings were consistent with the primary analysis and would lead to the same conclusions about the relative safety of antepartum nonindicated CD compared to SVD and intrapartum CD. (S1 Table and S2 Table).

## Discussion

This analysis of 108,847 deliveries in 2011 in China found a 54.6% CD rate and that 20.8% of deliveries were non-indicated CD, rates higher than the 46.2% and 11.6% rates respectively from the 2008 WHO study [1]. Among all CD, 92.2% were primary CD, 65.0% done prior to labor, and 38.1% non-indicated. While “maternal request” was the most often coded reason for CD, this may reflect a physician recommendation for CD. In a recent Shanghai study, women who had “CDMR” were likely to have had a CD recommended by an obstetrician [9].

The multivariable adjusted associations between mode of delivery and short-term maternal and neonatal outcomes found non-indicated antepartum CD was as safe as SVD for all short term outcomes measured. Specifically, non-indicated antepartum CD was associated with lower risk of PPH and was not associated with other severe maternal outcomes (transfusion, hysterectomy, or VTE) compared to SVD. Non-indicated antepartum CD had lower likelihood of neonatal death, ICU admission rates, occurrence of low Apgar, and RDS, and was not associated with infection, HIEE, birth trauma, or meconium aspiration compared to SVD.

Compared with women with SVD, women with indicated intrapartum CD had decreased rates of low Apgar and neonatal death; but higher rates of meconium aspiration.

Many of our findings on the safety of antepartum non-indicated cesareans are consistent with prior international [7,8,10] and Chinese studies but there are some notable differences. A 2015 Chinese study by Liu compared CDMR and planned vaginal delivery at one Shanghai hospital over 7 years in singleton, term, vertex, non-anomalous deliveries [18], See comment in PubMed Commons below the same population as our sensitivity analysis.

In our study, short-term maternal outcomes are generally consistent with the ACOG [7,10] and UK [8] CDMR guideline reviews and the Liu study [18]. Antepartum non-indicated CD was associated with a lower risk of PPH compared to vaginal delivery and intrapartum CD and no change in surgical complications. In the Liu study, severe PPH was not decreased with CDMR. Due to data limitations in our study, we could not differentiate severe PPH from mild PPH. The overall low rate of maternal surgical complications in this study may be explained by the lower obesity rate in the study (17.4%, defined as BMI ≥ 28) [16] compared to the USA rate in 2011 (34.9% defined as BMI ≥ 30) [19] and the low repeat CD rate (< 8%) and virtually no VBACs in 2011 (n = 2). Length of stay and urinary incontinence were not measured in this study.

One short-term neonatal outcome in this study that differed from the ACOG [7,10] and UK [8] CDMR guidelines and the Liu study [18] was the absence of an increased risk of iatrogenic prematurity and neonatal respiratory morbidity (RDS) associated with non-indicated antepartum CD compared to SVD. This may be explained by routine early dating ultrasounds in China and criteria for elective delivery after 39 weeks that averts some neonatal short-term morbidity (ICU admission, RDS, low Apgar) with non-indicated antepartum CD. The percent of non-indicated CD done after 39 weeks gestation was 57.7% in Liu’s study compared to 72.9% in our study, showing variable adherence to the 39-week elective CD rule at different institutions in China.

The other neonatal findings are consistent with the NIH [10] review including: a decrease in neonatal mortality, lack of association with neonatal infection rates, HIEE, and birth trauma for non-indicated CD compared to SVD, as is the higher infection, ICU admission, RDS, and meconium aspiration syndrome rates associated with intrapartum CD compared to pre-labor non-indicated CD and SVD.

A strength of the current study is the large number of deliveries and range of hospitals and regions. There are a substantial number of antepartum non-indicated CD for comparison to SVD and intrapartum CD.

There are some limitations of this study. We had limited data on socio-demographic variables such as income and compliance with prenatal care. Independent validation of the accuracy of the data collected was not done. The nonrandom selection of hospitals limits national generalizability in China. In this study, maternal mortality rate (MMR) of 6.4/100,00 is low likely due to under reporting and is not reliable. According to the Ministry of Health the national MMR in 2011 was 26.1/100,00 [20] and the Shanghai MMR was 9.6/100,000 [21]. In contrast the neonatal mortality rate (NNMR) seems more in-line with published data. In this study the neonatal mortality rate of 3.8/1000 reflects only early neonatal mortality and can be compared to national 7.8/1000 NNMR, which includes birth to 28 days [20]. If more clinical data were available many diagnoses included in the “indicated” CD category (oligohydramnios, suspected macrosomia, preeclampsia, third trimester bleeding, twins) could be re-categorized as non-indicated. This suggests our “non-indicated” CD rate is an underestimation.

This study looked at short-term outcomes in the birth hospitalization and information on readmission was not available. It does not address any potential long-term childhood issues associated with elective antepartum non-indicated CD such as obesity and childhood illness (asthma, diabetes) [22]. The epigenetic consequences and changes in microbiome associated with non-indicated CD need further investigation. Maternal morbidity and mortality beyond the hospital stay was not considered.

The USA [7] and UK [8] CDMR guidelines stress that non-indicated CD should not be motivated by fear of labor or unavailability of effective pain management and emotional support in labor. In China, in 2011 family presence in labor, nursing support and epidural anesthesia were not routinely available in public hospitals [23] but this study could not measure the effect of those factors on mode of delivery.

Prior reviews found the biggest risk of non-indicated CD in subsequent pregnancies with uterine rupture, placenta previa-accreta, and need for gravid hysterectomy [10]. The One Child Policy (1979–2015) regulated family size, resulting in few repeat CD and higher order pregnancies. In this 2011 study, 81.4% of the women were having their first birth and less than 8% of deliveries were to women with a prior CD. Thus, the morbidity and mortality associated with subsequent pregnancies after primary CD was not observed. With the new 2016 Two Child Policy in China, it is estimated that 90 million couples in China are now entitled to have a second child, that 25% will decide to have a second child, and many of these have had a non-indicated CD for the first delivery [24]. If a woman has a placenta previa and one prior cesarean, the risk of placenta accreta is 11–24% [25]. The estimated rate of uterine rupture with trial of labor (TOL) is 0.4 percent in women with a single prior low transverse CD [26]. The Chinese maternity care system will be challenged as more women have repeat CDs or trials of labor. Programs aimed at the safe prevention of primary CD in China may involve governmental regulation, evidence-based physician protocols, financial deterrents, and public education.

## Conclusion

In 2011, during the One Child Policy in China, with a skewed primiparous population, scheduled non-indicated CD was associated with short-term maternal and neonatal outcomes that were as safe or safer than vaginal delivery, while intrapartum CD was associated with increased maternal risks and minimal change in neonatal risk compared to SVD. In 2016, with the end of this policy and more women having a second child, the safety of non-indicated CD will likely change because evaluation of the world’s literature shows that the biggest risk of non-indicated CD is in subsequent pregnancies.

## Supporting information

S1 Appendix Approval from the human ethics committees received from hospitals. (DOCX)

S1 Table Associations between mode of delivery and maternal outcomes after excluding multiparous women, multiple gestations, mal-presentations, malformations, and preterm births. (DOCX)

S2 Table Associations between mode of delivery and neonatal outcomes after excluding multiparous women, multiple gestations, mal-presentations, malformations, and preterm births. (DOCX)
