# Supplementary material for: Cross sectional study of mode of delivery and maternal and perinatal outcomes in mainland China
Source: PLoS One. 2017 Feb 9;12(2):e0171779. doi: 10.1371/journal.pone.0171779 (PMC5300189; doi:10.1371/journal.pone.0171779)
Supplement: S1 Table — (DOCX) [file pone.0171779.s002.docx]

S1 Table. Associations between mode of delivery and maternal outcomes after excluding multiparous women, multiple gestations, mal-presentations, malformations, and preterm births.

| Mode of delivery | No outcome | Outcome | Crude | Adjusted* | Adjusted* |
| --- | --- | --- | --- | --- | --- |
|  | N (%) | N (%) | OR (99% CI) | OR (99% CI) | p-value |
| Outcome: Inpatient maternal death | | | | | |
| Spontaneous | 35819 (45.4%) | 1 (33.3%) | 1.00 |  |  |
| Operative vaginal delivery | 1073 (1.4%) | 0 (0%) | -- |  |  |
| Non-Indicated antepartum | 17245 (21.9%) | 0 (0%) | -- |  |  |
| Non-Indicated intrapartum | 2872 (3.6%) | 0 (0%) | -- |  |  |
| Indicated antepartum | 10101 (12.8%) | 2 (66.7%) | 7.09 (0.30, 166.3) |  |  |
| Indicated intrapartum | 11749 (14.9%) | 0 (0%) | -- |  |  |
| Outcome: Postpartum hemorrhage | | | | | |
| Spontaneous | 34741 (45.6%) | 1086 (39.7%) | 1.00 | 1.00 |  |
| Operative vaginal delivery | 991 (1.3%) | 82 (3.0%) | 2.65 (1.95, 3.60) | 2.32 (1.83, 2.93) | <0.0001 |
| Non-Indicated antepartum | 16862 (22.1%) | 384 (14.1%) | 0.73 (0.62, 0.85) | 0.71 (0.63, 0.80) | <0.0001 |
| Non-Indicated intrapartum | 2762 (3.6%) | 111 (4.1%) | 1.29 (0.99, 1.67) | 1.17 (0.96, 1.43) | 0.13 |
| Indicated antepartum | 9654 (12.7%) | 450 (16.5%) | 1.49 (1.29, 1.73) | 1.12 (0.99, 1.26) | 0.07 |
| Indicated intrapartum | 11130 (14.6%) | 620 (22.7%) | 1.78 (1.56, 2.04) | 1.51 (1.36, 1.67) | <0.0001 |
| Outcome: Maternal death, hysterectomy, transfusion, or VTE | | | | | |
| Spontaneous | 35739 (45.5%) | 88 (31.4%) | 1.00 | 1.00 |  |
| Operative vaginal delivery | 1063 (1.4%) | 10 (3.6%) | 3.82 (1.61, 9.06) | 3.58 (1.50, 8.55) | 0.0002 |
| Non-Indicated antepartum | 17210 (21.9%) | 36 (12.9%) | 0.85 (0.51, 1.42) | 0.84 (0.50, 1.40) | 0.37 |
| Non-Indicated intrapartum | 2861 (3.6%) | 12 (4.3%) | 1.70 (0.77, 3.77) | 1.62 (0.73, 3.62) | 0.12 |
| Indicated antepartum | 10054 (12.8%) | 50 (17.9%) | 2.02 (1.28, 3.19) | 1.01 (0.61, 1.68) | 0.96 |
| Indicated intrapartum | 11666 (14.8%) | 84 (30.0%) | 2.92 (1.97, 4.34) | 1.90 (1.25, 2.91) | <0.0001 |

*Adjusted for maternal age and education, gestational weeks, malpresentation, parity, multiple, placenta previa, placenta abruption, prebirth bleeding, medical complications, and malformation.
