# Supplementary material for: Cross sectional study of mode of delivery and maternal and perinatal outcomes in mainland China
Source: PLoS One. 2017 Feb 9;12(2):e0171779. doi: 10.1371/journal.pone.0171779 (PMC5300189; doi:10.1371/journal.pone.0171779)
Supplement: S2 Table — (DOCX) [file pone.0171779.s003.docx]

S2 Table. Associations between mode of delivery and neonatal outcomes after excluding multiparous women, multiple gestations, mal-presentations, malformations, and preterm births.

| Mode of delivery | No outcome | Outcome | Crude | Adjusted* | Adjusted* | |
| --- | --- | --- | --- | --- | --- | --- |
|  | N (%) | N (%) | OR (99% CI) | OR (99% CI) | p-value | |
| Outcome: Neonatal death | | | | | | |
| Spontaneous | 35731 (45.4%) | 83 (81.4%) | 1.00 | 1.00 |  | |
| Operative vaginal delivery | 1071 (1.4%) | 2 (2.0%) | 0.80 (0.13, 5.09) | 0.48 (0.05, 4.70) | 0.40 | |
| Non-Indicated antepartum | 17241 (21.9%) | 1 (1.0%) | 0.03 (0.00, 0.33) | 0.02 (0.00, 0.33) | 0.0002 | |
| Non-Indicated intrapartum | 2873 (3.6%) | 0 (0%) | -- | -- |  | |
| Indicated antepartum | 10095 (12.8%) | 7 (6.9%) | 0.30 (0.11, 0.82) | 0.13 (0.04, 0.39) | <0.0001 | |
| Indicated intrapartum | 11740 (14.9%) | 9 (8.8%) | 0.33 (0.13, 0.82) | 0.18 (0.07, 0.50) | <0.0001 | |
| Outcome: Admission to neonatal ICU | | | | | | |
| Spontaneous | 35469 (45.3%) | 345 (54.8%) | 1.00 | 1.00 |  | |
| Operative vaginal delivery | 1053 (1.3%) | 20 (3.2%) | 1.95 (1.07, 3.55) | 3.03 (1.63, 5.64) | <0.0001 | |
| Non-Indicated antepartum | 17181 (22.0%) | 61 (9.7%) | 0.37 (0.26, 0.52) | 0.50 (0.35, 0.72) | <0.0001 | |
| Non-Indicated intrapartum | 2855 (3.6%) | 18 (2.9%) | 0.65 (0.35, 1.21) | 0.80 (0.42, 1.51) | 0.35 | |
| Indicated antepartum | 10041 (12.8%) | 61 (9.7%) | 0.63 (0.44, 0.89) | 0.68 (0.47, 0.99) | 0.009 | |
| Indicated intrapartum | 11624 (14.9%) | 125 (19.8%) | 1.11 (0.84, 1.45) | 1.23 (0.93, 1.63) | 0.06 | |
| Outcome: Low Apgar（5min Apgar<4） | | | | | | |
| Spontaneous | 34172 (45.1%) | 47 (78.3%) | 1.00 | 1.00 |  | |
| Operative vaginal delivery | 1023 (1.4%) | 1 (1.7%) | 0.71 (0.05, 9.61) | 0.46 (0.02, 8.82) | 0.50 | |
| Non-Indicated antepartum | 16588 (21.9%) | 0 (0%) | -- | -- |  | |
| Non-Indicated intrapartum | 2836 (3.7%) | 0 (0%) | -- | -- |  | |
| Indicated antepartum | 9767 (12.9%) | 6 (10.0%) | 0.45 (0.15, 1.37) | 0.23 (0.06, 0.81) | 0.003 | |
| Indicated intrapartum | 11373 (15.0%) | 6 (10.0%) | 0.38 (0.13, 1.17) | 0.24 (0.07, 0.80) | 0.002 | |
| Outcome: Respiratory Distress (IRDS) | | | | | | |
| Spontaneous | 35720 (45.4%) | 95 (54.9%) | 1.00 | 1.00 |  | |
| Operative vaginal delivery | 1065 (1.4%) | 8 (4.6%) | 2.82 (1.09, 7.32) | 3.06 (1.17, 7.99) | 0.003 | |
| Non-Indicated antepartum | 17232 (21.9%) | 11 (6.4%) | 0.24 (0.11, 0.55) | 0.27 (0.12, 0.62) | <0.0001 | |
| Non-Indicated intrapartum | 2871 (3.6%) | 2 (1.2%) | 0.26 (0.04, 1.65) | 0.30 (0.05, 1.90) | 0.09 | |
| Indicated antepartum | 10072 (12.8%) | 30 (17.3%) | 1.12 (0.65, 1.92) | 1.18 (0.67, 2.07) | 0.45 | |
| Indicated intrapartum | 11722 (14.9%) | 27 (15.6%) | 0.87 (0.49, 1.52) | 0.92 (0.52, 1.63) | 0.71 | |
| Outcome: Infection | | | | | | |
| Spontaneous | 35802 (45.4%) | 13 (33.3%) | 1.00 | 1.00 | |  |
| Operative vaginal delivery | 1073 (1.4%) | 0 (0%) | -- | -- | |  |
| Non-Indicated antepartum | 17238 (21.9%) | 5 (12.8%) | 0.80 (0.21, 3.10) | 0.84 (0.21, 3.30) | | 0.74 |
| Non-Indicated intrapartum | 2872 (3.6%) | 1 (2.6%) | 0.96 (0.07, 13.9) | 0.98 (0.07, 14.5) | | 0.99 |
| With-indication antepartum | 10096 (12.8%) | 6 (15.4%) | 1.64 (0.46, 5.84) | 1.51 (0.41, 5.61) | | 0.42 |
| With-indication intrapartum | 11735 (14.9%) | 14 (35.9%) | 3.29 (1.22, 8.86) | 3.24 (1.19, 8.84) | | 0.003 |
| Outcome: HIEE/cerebral hemorrhage | | | | | | |
| Spontaneous | 35804 (45.4%) | 11 (68.8%) | 1.00 | 1.00 | |  |
| Operative vaginal delivery | 1072 (1.4%) | 1 (6.3%) | 3.04 (0.21, 44.8) | 8.40 (0.53, 133.8) | | 0.05 |
| Non-Indicated antepartum | 17243 (21.9%) | 0 (0%) | -- | -- | |  |
| Non-Indicated intrapartum | 2872 (3.6%) | 1 (6.3%) | 1.13 (0.08, 16.7) | 2.07 (0.14, 31.2) | | 0.49 |
| Indicated antepartum | 10101 (12.8%) | 1 (6.3%) | 0.32 (0.02, 4.75) | 0.46 (0.03, 7.32) | | 0.47 |
| Indicated intrapartum | 11747 (14.9%) | 2 (12.5%) | 0.55 (0.08, 4.02) | 0.76 (0.10, 5.64) | | 0.73 |
| Outcome: Birth Trauma | | | | | | |
| Spontaneous | 35800 (45.4%) | 15 (75.0%) | 1.00 | 1.00 | |  |
| Operative vaginal delivery | 1073 (1.4%) | 0 (0%) | -- | -- | | -- |
| Non-Indicated antepartum | 17241 (21.9%) | 2 (10.0%) | -- | -- | | -- |
| Non-Indicated intrapartum | 2873 (3.6%) | 0 (0%) | -- | -- | | -- |
| Indicated antepartum | 10100 (12.8%) | 2 (10.0%) | 0.32 (0.02, 4.75) | 0.43 (0.03, 6.76) | | 0.43 |
| Indicated intrapartum | 11748 (14.9%) | 1 (5.0%) | 0.55 (0.08, 4.02) | 0.72 (0.10, 5.37) | | 0.68 |
| Outcome: Meconium aspiration | | | | | | |
| Spontaneous | 35750 (45.4%) | 65 (44.8%) | 1.00 | 1.00 | |  |
| Operative vaginal delivery | 1068 (1.4%) | 5 (3.4%) | 2.58 (0.78, 8.53) | 3.24 (0.96, 10.9) | | 0.01 |
| Non-Indicated antepartum | 17226 (21.9%) | 17 (11.7%) | 0.54 (0.27, 1.10) | 0.70 (0.34, 1.42) | | 0.19 |
| Non-Indicated intrapartum | 2867 (3.6%) | 6 (4.1%) | 1.15 (0.38, 3.46) | 1.40 (0.46, 4.25) | | 0.44 |
| Indicated antepartum | 10092 (12.8%) | 10 (6.9%) | 0.55 (0.23, 1.31) | 0.54 (0.22, 1.34) | | 0.08 |
| Indicated intrapartum | 11707 (14.9%) | 42 (29.0%) | 1.97 (1.18, 3.29) | 2.01 (1.18, 3.40) | | 0.0007 |
